# Hypersensitive C-reactive protein-albumin ratio predicts symptomatic intracranial hemorrhage after endovascular therapy in acute ischemic stroke patients

**DOI:** 10.1186/s12883-021-02066-2

**Published:** 2021-02-01

**Authors:** Qiang Peng, Jiankang Hou, Siyu Wang, Feng Zhou, Yan E, Wei Wang, Ting Huang, Meng Wang, Shi Huang, Junshan Zhou, Nihong Chen, Yingdong Zhang

**Affiliations:** 1 Department of Neurology, Nanjing First Hospital, Nanjing Medical University, No. 68 Changle Road, Nanjing, 210006 P.R. China; 2 Department of Neurology, Nanjing Yuhua Hospital, Nanjing First Hospital, Nanjing Medical University, Nanjing, 210006 Jiangsu China

**Keywords:** Hypersensitive C-reactive protein-albumin ratio, Acute ischemic stroke, Endovascular therapy, Symptomatic intracranial hemorrhage, Risk factor

## Abstract

**Background:**

Approximately 10% of patients would develop symptomatic intracranial hemorrhage (sICH) after endovascular therapy. The aim of our study was to explore the ability of hypersensitive C-reactive protein-albumin ratio (HAR) in predicting sICH after endovascular therapy.

**Methods:**

From April 2016 to December 2018, 334 consecutive patients with anterior circulation infarction undergoing endovascular therapy were enrolled in our study. sICH was defined using Heidelberg bleeding classification after endovascular therapy. Multiple regression analysis was used to investigate the potential risk factors of sICH after endovascular therapy. We used receiver operating characteristic curve analysis and nomogram analysis to assess the overall discriminative ability of the HAR in predicting sICH after endovascular therapy.

**Results:**

Among these 334 patients enrolled, 37 (11.1%) patients with anterior circulation infarction were identified with sICH after endovascular therapy. Univariate logistic regression analysis demonstrated that patients with higher levels of HAR may be inclined to develop sICH (odds ratio, 10.994; 95% confidence interval, 4.567–26.463; *P* = 0.001). This association remained significant even after adjustment for potential confounders. Also, a cutoff value of 0.526× 10^− 3^ for HAR was detected in predicting sICH (area under curve, 0.763). Furthermore, nomogram analysis also suggested that HAR was an indicator of sICH (c-index was 0.890, *P*< 0.001).

**Conclusions:**

This study showed that high levels of HAR could predict sICH after endovascular therapy.

**Supplementary Information:**

The online version contains supplementary material available at 10.1186/s12883-021-02066-2.

## Background

Stroke is one of leading causes of death and long-term disability globally [1]. According to several randomized controlled trials focusing on acute ischemic stroke because of large vessel occlusion in the anterior circulation, endovascular treatment has been demonstrated benefits in abating mortality and morbidity of stroke patients [2–6]. Nevertheless, some patients may develop symptomatic intracranial hemorrhage (sICH) within 24 h after endovascular therapy, which has been reported to be associated with symptoms worsening as well as poor outcomes [7, 8]. Therefore, it is notable for neurologists to explore the risk factors and predictors of sICH after endovascular therapy.

It is reported that inflammation may be related to central nervous system diseases, such as stroke, Parkinson’s Disease and Alzheimer disease, and plays a crucial role in the mechanism [9–12]. Gong and his colleagues found the association between high-sensitivity C-reactive protein at admission and progressive motor deficits in patients with penetrating artery infarctions [13]. Previous studies also discovered that albumin might be able to attenuate excessive innate immunity in subarachnoid hemorrhage [14, 15]. High-sensitivity C-reactive protein to albumin ratio (HAR), a novel biomarker which combines high-sensitivity C-reactive protein and albumin, was reported to have to do with left ventricular thrombus formation following acute anterior myocardial infarction [16]. According to a recent study, increased serum HAR may be independently associated with the severity of carotid artery stenosis [17]. Moreover, the relationship between C-reactive protein to albumin ratio and 90-day mortality in patients with acute ischemic stroke has been confirmed [18].

However, the correlation between HAR and sICH after endovascular treatment has not been clarified. Hence, this observational study was performed to explore the predictive utility of HAR in sICH after endovascular treatment, prospectively.

## Methods

### Study population

This prospective study was performed from April 2016 to December 2018 in Nanjing First Hospital, Nanjing Medical University. Acute ischemic stroke patients with anterior circulation infarction, who were treated with endovascular therapy within 6 h after symptom onset, were included in the study. The patients treated with a bridging therapy, which consists of intravenous thrombolysis and endovascular therapy, were also included. The exclusion criteria were as follows: (1) age less than 18 years; (2) posterior circulation infarction; (3) systemic inflammatory disease, renal failure, hepatic failure, brain tumor and presence of an active infection. Informed consent was obtained from participants or legal representatives, and the protocol was approved by the Ethical Committee of Nanjing First Hospital, Nanjing Medical University.

### Clinical assessments

Clinical assessments were performed within 24 h after admission. All participants had standard assessments of demographic characteristics (including age and sex), vascular risk factors (including hypertension, diabetes mellitus, dyslipidemia, previous stroke, atrial fibrillation, coronary heart disease, current smoking and current drinking), previous antiplatelet, systolic blood pressure (SBP) at admission, diastolic blood pressure (DBP) at admission, time from door to puncture, intravenous thrombolysis, number of thrombectomy device passes, stroke severity, stroke subtype, and laboratory data. Computed tomography, magnetic resonance, digital subtraction angiography, electrocardiogram, transcranial Doppler and carotid ultrasonography were performed for assessing the stroke etiology. Stroke subtype was classified according to Trial of Org 10,172 in Acute Stroke Treatment (TOAST) criteria [19]. Successful recanalization was defined as a TICI score of 2b-3 [20]. Collateral status was assessed based on digital subtracted angiography using the American Society of Interventional and Therapeutic Neuroradiology/Society of Interventional Radiology grading system, with grade 0 to 1 representing poor collateral status and grade 2 to 4 representing moderate to excellent [21]. The blood samples to measure the levels of Hs-CRP and albumin were collected at 7 AM the next day after admission. The measurement of Hs-CRP and albumin was completed within 24 h after admission.

### Definition of sICH

Symptomatic Intracranial Hemorrhage (sICH) was defined as any hemorrhagic transformation associated with total NIHSS score worsening ≥ 4 points or worsening ≥2 points in one NIHSS category or deterioration led to intubation, hemicraniectomy, external ventricular drain placement, or any other major interventions, which used the Heidelberg bleeding classification [7, 22, 23]. The evaluation of neurological deficits was conducted using National Institutes of Health Stroke Scale (NIHSS) score on admission and continued the following 24 h after endovascular therapy by two certified neurologists blind to clinical data. In case of disagreement about the NIHSS score evaluation, a third neurologist was invited for a final decision.

### Measurement of laboratory data

All the blood samples were collected at 7 AM the next day after admission. The levels of Hs-CRP were measured with an immunoturbidimetry assay by an Architect c16000 chemistry analyzer (Abbott Diagnostics, Chicago, USA). The levels of albumin were measured with a chemiluminescence detection assay by an AU5800 chemistry analyzer (Beckman Coulter, Brea, USA).

### Statistical analysis

Statistical analyses were performed with R version 3.6.2 software (http://www.R-project.org/). Categorical variables were expressed as n (%) and continuous variables were expressed as means (standard deviation, SD) or medians (interquartile range, IQR). Differences in baseline characteristics between groups were analyzed using independent sample t-tests or Mann-Whitney U tests for continuous variables as well as the Chi-squared test or Fisher’s exact test for categorical variables, as appropriate. Multivariable analysis was adjusted for all potential confounders with statistically significant association at *P* < 0.05 in univariate regression analysis. Receiver operating characteristic (ROC) curve analysis was performed to assess the overall discriminative ability of the HAR to predict sICH after endovascular therapy and to establish optimal cutoff points at which the sum of the specificity and sensitivity was the highest. A MedCalc 15.6.0 (MedCalc Software Acacialaan 22, B-8400 Ostend, Belgium) packet program was used to obtain ROC. A nomogram based on the independent predictors was constructed by R software with the package rms. The predictive capacity of the nomogram was determined by Harrell’s c-index. A two-tailed value of *P* < 0.05 was considered significant.

## Results

From April 2016 to December 2018, five hundred and twelve patients were enrolled in this study. One hundred and seventy-eight patients were excluded for the reasons below: posterior circulation infarct (*n*=149), systemic inflammatory disease (*n*=3), renal failure (*n*=16), hepatic failure (*n*= 4), brain tumor (n=1) and presence of an active infection (*n*=5). A total of 334 subjects (210 men; mean age, 69.0 ± 11.2 years) were included for the final analysis. Among these patients, 229 (68.6%) had hypertension, 67 (20.1%) had diabetes mellitus, 100 (29.9%) had dyslipidemia, 75 (22.5%) had coronary heart disease, and 99 (29.6%) had atrial fibrillation.

Among the 334 patients enrolled, sICH was observed in 37 patients (11.1%). Table 1 showed the comparisons of baseline characteristics in patients with or without sICH after endovascular therapy. Compared to the patients without sICH after endovascular therapy, those patients with sICH were older (*P* = 0.016) and had higher proportions of hypertension (*P* = 0.014) as well as previous antiplatelet use (*P*=0.003); Higher levels of SBP (*P* = 0.017), initial NIHSS score (*P* = 0.043), the number of thrombectomy device passes (*P* = 0.048), fasting blood glucose (*P* = 0.001) and HAR (*P* = 0.001).

**Table 1:** Characteristics of subgroups based on the presence of sICH

Variable	sICH group (*n*=37)	Non-sICH group (*n*=297)	*P*
Demographic characteristics			
Age, years	73.2 ±10.9	68.5 ±11.2	0.016
Male, %	23 (62.2)	187 (63.0)	0.904
Vascular risk factors, %			
Hypertension	32 (86.5)	197 (66.3)	0.014
Diabetes mellitus	9 (24.3)	58 (19.5)	0.515
Dyslipidemia	7 (18.9)	93 (31.3)	0.132
Coronary heart disease	12 (32.4)	63 (21.2)	0.529
Atrial fibrillation	14 (37.8)	85 (28.6)	0.076
Previous stroke	9 (24.3)	70 (23.6)	0.100
Current drinking	8 (21.6)	82 (27.6)	0.421
Current smoking	9 (24.3)	102 (34.3)	0.144
Clinical data			
Previous antiplatelet, %	14 (37.8)	47 (15.8)	0.003
SBP, mmHg	149.7 ±22.7	139.8 ±23.9	0.017
DBP, mmHg	84.1 ±17.7	85.2 ±14.6	0.668
Body mass index, kg/m^2^	24.2 ±3.8	24.1 ±3.7	0.919
Initial NIHSS, score	16 (13, 22)	15 (11, 19)	0.043
Intravenous thrombolysis, %	21 (56.8)	122 (41.1)	0.069
Time from door to puncture, min	120.0 (90.5, 149.0)	110.0 (75.0, 140.0)	0.075
Number of thrombectomy device passes	2 (1, 3)	2 (1, 3)	0.048
Poor collateral status, %	23 (62.2)	135 (45.5)	0.055
Successful recanalization, %	33 (89.2)	256 (86.2)	0.800
Stroke subtype, %			0.169
LAA	12 (32.4)	139 (46.8)	
CE	21 (56.8)	121 (40.7)	
Other subtype	4 (10.8)	37 (12.5)	
Laboratory data			
TC, mmol/L	4.2 ±1.2	4.4 ±1.2	0.537
TG, mmol/L	1.1 (0.8, 1.7)	1.1 (0.8, 1.6)	0.375
LDL, mmol/L	2.6 (1.9, 3.2)	2.6 (2.0, 3.2)	0.469
HDL, mmol/L	1.1±0.3	1.1±0.4	0.865
FBG, mmol/L	9.0 ±3.5	6.9 ±2.4	0.001
Homocysteine, μmol/L	12.8 (10.7, 16.0)	12.3 (10.4, 15.8)	0.588
HAR, 10^−3^	0.54 (0.24, 1.55)	0.17 (0.09, 0.33)	0.001

Abbreviation: *NIHSS*, National institute of health stroke scale, *SBP* Systolic blood pressure, *DBP* Diastolic blood pressure, *LAA* Large-artery atherosclerosis, *CE* Cardioembolism, *TC* Total cholesterol, *TG* Triglyceride, *LDL* Low-density lipoprotein, *HDL* High-density lipoprotein, *FBG* Fasting blood glucose, *HAR* Hypersensitive C-reactive protein-albumin ratio

Table 2 showed the results of logistic regression analysis for risk factors with sICH after endovascular therapy. Univariate logistic regression analysis exhibited that HAR [odds ratio (OR), 10.994; 95% confidence interval (CI) 4.567–26.463, *P* = 0.001], age (OR, 1.044; 95% CI 1.008–1.081, *P*= 0.017), hypertension (OR, 3.249; 95% CI 1.228–8.594, *P*= 0.018), previous antiplatelet use (OR, 3.225; 95% CI 1.548–6.718, *P*= 0.002), SBP (OR, 1.017; 95% CI 1.003–1.031, *P*= 0.019), initial NIHSS score (OR, 1.058; 95% CI 1.011–1.107, *P*= 0.014), the number of thrombectomy device passes (OR, 1.362; 95% CI 1.046–1.722, *P*= 0.022) and fasting blood glucose level (OR, 1.273; 95% CI 1.140–1.421, *P*= 0.001) might be associated with sICH after endovascular therapy. After adjustment for all potential confounders, HAR was identified as an independent risk factor for sICH after endovascular therapy (OR, 12.384; 95% CI 4.379–35.023, *P*= 0.001). Moreover, fasting blood glucose level (OR, 1.333; 95% CI 1.145–1.552, *P*= 0.001) and previous antiplatelet use (OR, 4.940; 95% CI 1.920–12.713, *P*=0.001) were also related to sICH, independently.

**Table 2:** Logistic regression analysis for risk factors with symptomatic intracranial hemorrhage after endovascular therapy

Variable	Unadjusted OR (95%CI)	*P*	Adjusted OR (95%CI)	*P*
Demographic characteristics				
Age, years	1.044 (1.008–1.081)	0.017	1.028 (0.984–1.074)	0.215
Male	0.958 (0.473–1.938)	0.904		
Vascular risk factors				
Hypertension	3.249 (1.228–8.594)	0.018	3.478 (0.956–12.657)	0.059
Diabetes mellitus	1.325 (0.593–2.959)	0.493		
Dyslipidemia	0.512 (0.217–1.208)	0.126		
Coronary heart disease	1.783 (0.849–3.746)	0.127		
Atrial fibrillation	1.518 (0.746–3.089)	0.249		
Previous stroke	1.038 (0.467–2.304)	0.927		
Current drinking	0.720 (0.316–1.640)	0.434		
Current smoking	0.661 (0.278–1.345)	0.221		
Clinical data				
Previous antiplatelet	3.225 (1.548–6.718)	0.002	4.940 (1.920–12.713)	0.001
SBP	1.017 (1.003–1.031)	0.019	1.012 (0.995–1.030)	0.174
DBP	0.995 (0.972–1.018)	0.667		
Body mass index	1.005 (0.916–1.102)	0.919		
Initial NIHSS	1.058 (1.011–1.107)	0.014	1.033 (0.970–1.101)	0.306
Intravenous thrombolysis	1.883 (0.944–3.755)	0.072		
Time from door to puncture	1.002 (0.998–1.006)	0.329		
Number of thrombectomy device passes	1.362 (1.046–1.772)	0.022	1.396 (0.982–1.985)	0.063
Poor collateral status	1.971 (0.976–3.980)	0.058		
Successful recanalization	1.321 (0.445–3.925)	0.616		
Stroke subtype, %				
LAA	Reference			
CE	0.799 (0.243–2.620)	0.711		
Other subtype	1.605 (0.518–4.974)	0.412		
Laboratory data				
TC	0.906 (0.663–1.238)	0.535		
TG	1.129 (0.831–1.532)	0.438		
HDL	0.917 (0.338–2.487)	0.865		
LDL	0.906 (0.621–1.322)	0.609		
FBG	1.273 (1.140–1.421)	0.001	1.333 (1.145–1.552)	0.001
Homocysteine	1.000 (0.999–1.001)	0.865		
HAR	10.994 (4.567–26.463)	0.001	12.384 (4.379–35.023)	0.001

Abbreviation: *NIHSS*, National institute of health stroke scale, *SBP* Systolic blood pressure, *DBP* Diastolic blood pressure, *LAA* Large-artery atherosclerosis, *CE* Cardioembolism, *TC* Total cholesterol, *TG* Triglyceride, *LDL* Low-density lipoprotein, *HDL* High-density lipoprotein, *FBG* Fasting blood glucose, *HAR* Hypersensitive C-reactive protein-albumin ratio

**Fig. 1:**
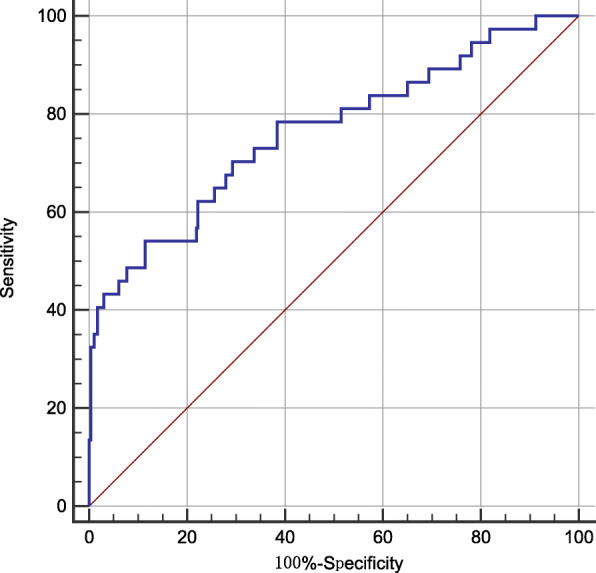
Receiver operating characteristic (ROC) curve for the value of HAR to predict sICH

Figure 1 exhibited the ROC curve, which showed the predictive ability of HAR in sICH after endovascular therapy. The optimal cutoff value for HAR as a predictor of sICH was determined as 0.526× 10^− 3^ in the ROC curve analysis, which yielded a sensitivity of 54.1% and a specificity of 88.6%, with the AUC at 0.763 (95% CI, 0.714–0.808). What is more, we also compared the ROC of HAR and Hs-CRP. The AUCs of HAR and Hs-CRP are 0.763 and 0.718, respectively. According to Z test about pairwise comparison of ROC curves, HAR is able to predict better than Hs-CRP alone (*P* = 0.0003), which can be seen in the Supplementary Figure 1 and Supplementary Figure 2.

**Fig. 2:**
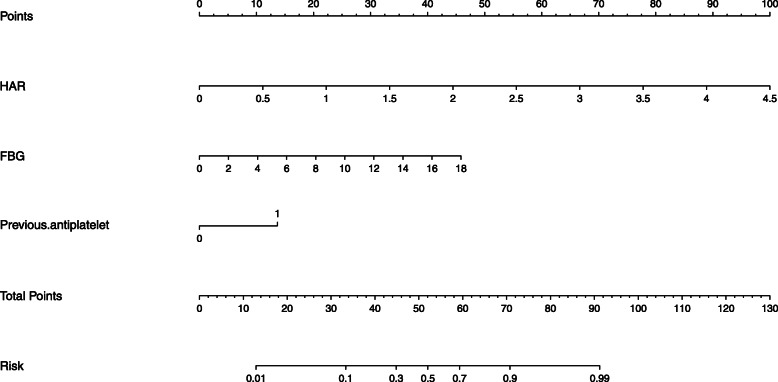
The nomogram of patients treated with endovascular therapy for predicting sICH

Figure 2 showed the nomogram. The concordance index was 0.890 (*P*< 0.001). The novel model indicated that higher HAR was an independent indicator of sICH after endovascular therapy. These findings were similar to those findings above, which was obtained previously in the multivariate logistic model and ROC curve analysis.

## Discussion

This observational research helped us find the relationship between HAR and sICH after endovascular treatment. The anterior circulation infarction patients undergoing endovascular treatment with elevated levels of HAR are prone to develop sICH. After the adjustment for fasting blood glucose level, previous antiplatelet use and some other variables, HAR remains a significant independent risk factor of sICH after endovascular treatment. It is widely believed that one biomarker with 0.7 < area under the curve < 0.9 presents a moderate diagnostic value [9, 24]. Therefore, HAR might serve as a biomarker associated with sICH after endovascular treatment. Furthermore, the nomogram about sICH after endovascular treatment confirmed this conclusion.

In our study, the incidence of sICH after endovascular treatment is 11.1%, which is consistent with previous studies [23, 25]. This may be relevant to the rigorous evaluation in our stroke center. HAR has been reported to be related to the severity of carotid artery stenosis, which is related to the severity and prognosis of stroke [17]. A recent study showed that high levels of HAR at admission were significantly associated with intra-hospital mortality in the patients with intracerebral hemorrhage, another type of stroke [26]. Kocatürk M and colleagues also found the relationship between HAR and the prognosis in patients with acute stroke [18]. To our knowledge, this study is the first to investigate the prospective association of HAR with sICH after endovascular treatment, and reveals that HAR could act as a useful predictor for sICH in patients undergoing endovascular treatment, according to the results of multivariate logistic regression, ROC analysis as well as nomogram analysis. Our study also found that previous antiplatelet use and fasting blood glucose level are associated with sICH after endovascular treatment, which is in line with other researches [25, 27, 28].

The mechanisms underlying the association between HAR and sICH after endovascular treatment are not entirely clear, and further studies are warranted. In term of previous researches, inflammation plays an important role in the pathophysiological mechanism of ischemic stroke [29–32]. Oxidative stress and inflammatory activity could lead to blood brain barrier disruption, which may be linked to hemorrhagic transformation after endovascular treatment [33]. High sensitivity C-reactive protein is a crucial inflammatory marker. In response to IL-1, IL-6 and TNF-α, hepatocytes produced high sensitivity C-reactive protein in large amounts [34, 35]. Pro-inflammatory cytokine, might be involved in the pathophysiology of sICH after endovascular treatment through inflammatory responses, may be induced by high sensitivity C-reactive protein. In addition, several clinical studies also found that high-sensitivity C-reactive protein is associated with progressive stroke [9] and post-stroke fatigue [36]. Moreover, it is believed that albumin, a heart-shaped plasma protein with a 585 amino acid polypeptide chain, possesses neuroprotective effect, and serum albumin levels are likely lower in inflammatory states [37]. Albumin might serve as an anti-inflammatory agent and attenuate microglial and T cell activation [38]. Albumin administration has been proven to improve neurobehavior in experimental subarachnoid hemorrhage [15]. Nevertheless, only single biomarker might be affected by pathophysiological changes. HAR, the combination of high sensitivity C-reactive protein and albumin, may be able to provide more compound information in predicting sICH in patients undergoing endovascular treatment.

Although we have discovered the relationship between HAR and sICH after endovascular treatment, there are still some limitations in this clinical research. First, this study is an observational research, not randomized controlled trial. The sample size of our observational research is also relatively small. Second, this research was conducted in a single stroke center in China, so the conclusion is supposed to be verified in other stroke center to confirm that this finding can be extrapolated to other populations. Third, it is reported that several biomarkers may change during hospitalization [39]. Therefore, in further studies, we should examine the biomarker, such as high sensitivity C-reactive protein and albumin, dynamically. What is more, we do not have accurate data about the duration of the endovascular procedure, but we aim to evaluate them in our further studies to explore the relationship between the duration of the endovascular procedure and sICH after endovascular treatment. Despite the shortcomings above, this study has found the predictive value of HAR for sICH after endovascular treatment, which is helpful for exploring the mechanism of sICH after endovascular treatment.

## Conclusion

In summary, the results of our study indicated that the higher levels of HAR were associated with increasing risk of sICH after endovascular treatment. Moreover, HAR may be able to serve as a non-invasive predictor for sICH in patients with stroke undergoing endovascular treatment. More multicenter studies with large sample size are needed in the future. In addition, neurologists may pay more attention to the patients with higher levels of HAR after endovascular treatment in the future clinical practice.

## Supplementary Information

**Additional file 1. ** The comparison of ROC curves for sICH after endovascular therapy.

**Additional file 2. ** The details about the comparison of ROC curves for sICH after endovascular therapy.

## Data Availability

The data that support the findings of this study are available from the corresponding author upon reasonable request.
