# Supplementary figures and images for: Hypersensitive C-reactive protein-albumin ratio predicts symptomatic intracranial hemorrhage after endovascular therapy in acute ischemic stroke patients
Source: BMC Neurol. 2021 Feb 1;21:47. doi: 10.1186/s12883-021-02066-2 (PMC7849085; doi:10.1186/s12883-021-02066-2)

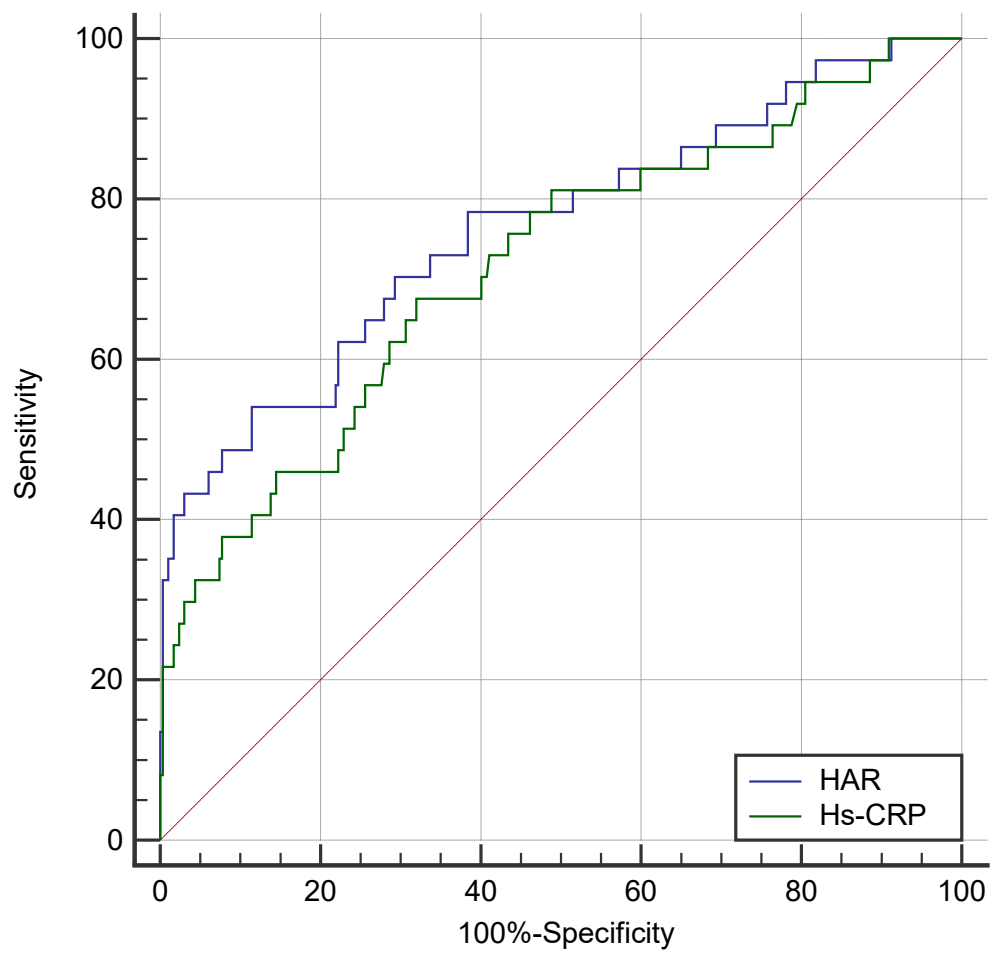

Supplement: Supplementary file 1 — Additional file 1. The comparison of ROC curves for sICH after endovascular therapy. [file 12883_2021_2066_MOESM1_ESM.pdf]
