# Supplementary material for: Hypersensitive C-reactive protein-albumin ratio predicts symptomatic intracranial hemorrhage after endovascular therapy in acute ischemic stroke patients
Source: BMC Neurol. 2021 Feb 1;21:47. doi: 10.1186/s12883-021-02066-2 (PMC7849085; doi:10.1186/s12883-021-02066-2)
Supplement: Supplementary file 2 — Additional file 2. The details about the comparison of ROC curves for sICH after endovascular therapy. [file 12883_2021_2066_MOESM2_ESM.pdf]

Comparison of ROC curves

Variable 1	HAR
Variable 2	Hs_CRP<br>Hs-CRP
Classification variable	sICH

Sample size	334
Positive group [a]	37 (11.08%)
Negative group [b]	297 (88.92%)

[a] sICH = 1
[b] sICH = 0

Variable	AUC	SE [a]	95% CI [b]
HAR	0.763	0.0481	0.714 to 0.808
Hs_CRP	0.718	0.0488	0.666 to 0.765

[a] DeLong et al., 1988
[b] Binomial exact

Pairwise comparison of ROC curves

HAR ~ Hs_CRP	
Difference between areas	0.0457
Standard Error [a]	0.0127
95% Confidence Interval	0.0209 to 0.0705
z statistic	3.610
Significance level	P = 0.0003

[a] DeLong et al., 1988
